# Natriuretic Peptide Receptor A as a Novel Target for Prostate Cancer

**DOI:** 10.1186/1476-4598-10-56

**Published:** 2011-05-17

**Authors:** Xiaoqin Wang, Payal Raulji, Shyam S Mohapatra, Ronil Patel, Gary Hellermann, Xiaoyuan Kong, Pedro L Vera, Katherine L Meyer-Siegler, Domenico Coppola, Subhra Mohapatra

**Affiliations:** 1 Department of Molecular Medicine, University of South Florida, Tampa FL, 33612, USA; 2 Department of Internal Medicine, University of South Florida, Tampa FL, 33612, USA; 3 Department of Surgery, University of South Florida, Tampa FL, 33612, USA; 4 Bay Pines Veterans Affairs Healthcare System, Bay Pines, FL 33744, USA; 5 H. Lee Moffitt Cancer Center; 6 James A. Haley Veterans Hospital, Tampa FL, 33612, USA

## Abstract

**Background:**

The receptor for the cardiac hormone atrial natriuretic peptide (ANP), natriuretic peptide receptor A (NPRA), is expressed in cancer cells, and natriuretic peptides have been implicated in cancers. However, the direct role of NPRA signaling in prostate cancer remains unclear.

**Results:**

NPRA expression was examined by western blotting, RT-PCR and immunohistochemistry. NPRA was downregulated by transfection of siRNA, shRNA and NPRA inhibitor (iNPRA). Antitumor efficacy of iNPRA was tested in mice using a TRAMP-C1 xenograft. Here, we demonstrated that NPRA is abundantly expressed on tumorigenic mouse and human prostate cells, but not in nontumorigenic prostate epithelial cells. NPRA expression showed positive correlation with clinical staging in a human PCa tissue microarray. Down-regulation of NPRA by siNPRA or iNPRA induced apoptosis in PCa cells. The mechanism of iNPRA-induced anti-PCa effects was linked to NPRA-induced expression of macrophage migration inhibitory factor (MIF), a proinflammatory cytokine over-expressed in PCa and significantly reduced by siNPRA. Prostate tumor cells implanted in mice deficient in atrial natriuretic peptide receptor A (NPRA-KO) failed to grow, and treatment of TRAMP-C1 xenografts with iNPRA reduced tumor burden and MIF expression. Using the TRAMP spontaneous PCa model, we found that NPRA expression correlated with MIF expression during PCa progression.

**Conclusions:**

Collectively, these results suggest that NPRA promotes PCa development in part by regulating MIF. Our findings also suggest that NPRA is a potential prognostic marker and a target for PCa therapy.

## Introduction

Prostate cancer (PCa) is the third leading cause of death among men in America [1,2]. The mortality from PCa results from metastases to bones and lymph nodes and progression from androgen-dependent to androgen-independent disease. While androgen deprivation has been effective in treating androgen-dependent PCa, it is ineffective in treating advanced PCas, the primary cause of mortality. Epidemiological and histopathological studies have implicated inflammation in the pathogenesis of PCa [3-5]. Studies have consistently shown a decreased risk of PCa among men who regularly take aspirin or other nonsteroidal anti-inflammatory drugs (NSAIDs) [6-8]. Despite beneficial effects, the side effects from using high doses of COX-2 inhibitors for cancer prevention are a major concern. These observations emphasize the need for development of new effective treatments for advanced PCa.

The family of natriuretic peptide hormones has broad physiologic effects. In addition to vasodilation, cardiovascular homeostasis, sodium excretion and inhibition of aldosterone secretion, they have been implicated in immunity and inflammation [9-18]. The effects of atrial natriuretic peptide (ANP) are mediated by its interaction with the cell surface natriuretic peptide receptor A (NPRA; high affinity) and natriuretic peptide receptor C (NPRC; low affinity). In patients with prostate tumors, the immune response plays a large part in the progression of the disease and it is likely that the NPRA system is involved; but the role of NPRA in human cancers remains unknown. A novel peptide, NP_73-102_, has been identified [14] whose sequence is immediately N-terminal to the ANP peptide and which is an inhibitor of NPRA (iNPRA). NP_73-102 _does not bind to NPRA but blocks its expression, and we have shown that it possesses bronchodilatory, anti-inflammatory [14,16,19,20] and antitumor activity [19].

We previously reported that mice deficient in NPRA (NPRA-knockout, KO) exhibit significantly decreased inflammation [16,19-21]. Furthermore, we found that NPRA-KO mice do not permit growth of implanted human lung cancer, melanoma and ovarian cancer cells [19], suggesting that NPRA may be a novel therapeutic candidate. Given the evolutionary conservation of ANP in many species, we reasoned that NPRA expression may be relevant in human cancers. In this study, we examined the expression of NPRA in PCa cell lines and human tissue samples and determined whether NPRA can be used as a target for PCa therapy. The results show that increased NPRA expression is strongly associated with progression of human PCa and that NPRA deficiency prevents growth of transplanted PCa cells and inhibits tumor burden in part by downregulating macrophage migration inhibitory factor (MIF) in PCa cells.

## Results

### PCa cells have increased NPRA levels

**Figure 1:**
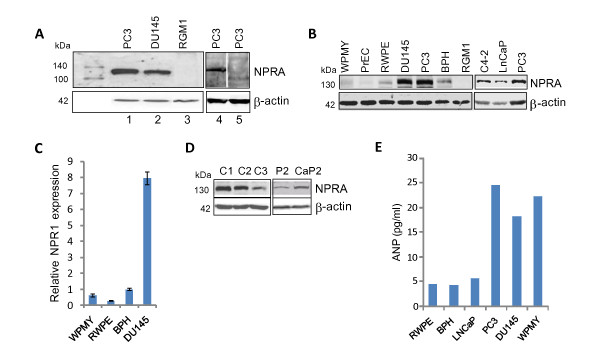
**NPRA expression in tumor cells**. (A) Immunoblot analysis demonstrating specificity of anti-NPRA antibody. Cell lysates were subjected to SDS-PAGE/immunoblot analysis with rabbit polyclonal antibody specific for human NPRA (top) and beta-actin (bottom). For the competition assay, lane 5 was incubated with NPRA-antibody adsorbed with NPRA peptide (20 μg/ml). (B) Western blotting for NPRA expression. β-actin expression was used as loading control. (C) Total RNA of indicated cell lines was analyzed for NPRA by real time PCR. NPRA expression was normalized with respect to β-actin. (D) Whole cell lysates of TRAMP-C1, C2, C3, CaP2 and P2 cells were analyzed by western blotting for NPRA. (E) ANP expression in PCa cells. Cells were cultured in media containing 0.5% fetal bovine serum for 48 hrs and the culture supernatants were analyzed for ANP by enzyme immunoassay kit (Phoenix Pharmaceuticals).

NPRA expression studies in human tissues have been limited by lack of availability of appropriate antibodies to NPRA. The antibodies that are commercially available are very poor in quality and do not provide consistent results. We developed an antibody to NPRA in rabbits using a specific antigenic peptide (amino acids 1010-1031 of mouse NPRA protein, which is homologous to rat and human NPRA). As shown in Figure 1A, an approximately 130 kDa band corresponding to NPRA was detected only in human PCa cell lines, PC3 and DU145 that express NPRA, but not in the RGM1 cell line that does not express NPRA [22]. The specificity of the anti-NPRA antibody was confirmed by ELISA (Additional file 1, Fig. S1A), western blotting (Figure 1A, lane 5) and by immunofluorescence (Additional file 1, Fig. S1B) and immunohistochemistry (Additional file 1 Fig.S1C-D).

We examined NPRA expression by western blotting in various types of PCa tumors and compared it with that in normal prostate epithelial cells (PrEC and RWPE) and benign prostatic hyperplasia (BPH) cells. Results of the western blot show that NPRA is expressed abundantly in the androgen-dependent PCa cell line, LNCaP and androgen-independent cell lines C4-2, PC3 and DU145, but not in PrEC cells and only weakly in RWPE and BPH cells (Figure 1B). Very little NPRA is detected in the stromal cell line, WPMY, which is derived from normal prostate. NPRA protein expression in DU145 cells correlated with mRNA level, as verified by real-time PCR (Figure 1C). Lysates of normal RGM1 cells that do not express NPRA were used as control. NPRA is also highly expressed in transplantable syngeneic tumor lines derived from TRAMP (transgenic adenocarcinoma mouse prostate) mice which get spontaneous PCa. NPRA is strongly expressed in the tumorigenic TRAMP-C1 and -C2 PCa cell lines but less abundantly in the non-tumorigenic TRAMP-C3 PCa cell line (Figure 1D) [23]; the latter shows a three-fold reduction in growth and colonization potential compared to TRAMP-C1 and C2 cells (Additional file 2, Fig. S2). In addition, increased NPRA expression was seen in prostate epithelial lines from intact conditional homozygous Pten knockout mice (PTEN-CaP2) that are tumorigenic compared to heterozygous Pten knockout mice (PTEN-P2) (Figure 1D) [24]. These results suggest that NPRA is more abundantly expressed in PCa cells than normal or benign prostate epithelial cells. Expression of the natural ligand for NPRA, ANP was examined in cultured PCa cells. ANP expression was detected in culture supernatants of PC3 and DU145 PCa cells and WPMY stromal cells (Figure 1E) but not in normal prostate epithelial cells or LNCaP cells. These results suggest that NPRA is predominantly expressed in prostate tumor cells, while ANP is expressed in stromal cells and in androgen-independent PCa cells, but not in androgen-dependent cells.

### NPRA protein expression correlates with human PCa progression

**Figure 2:**
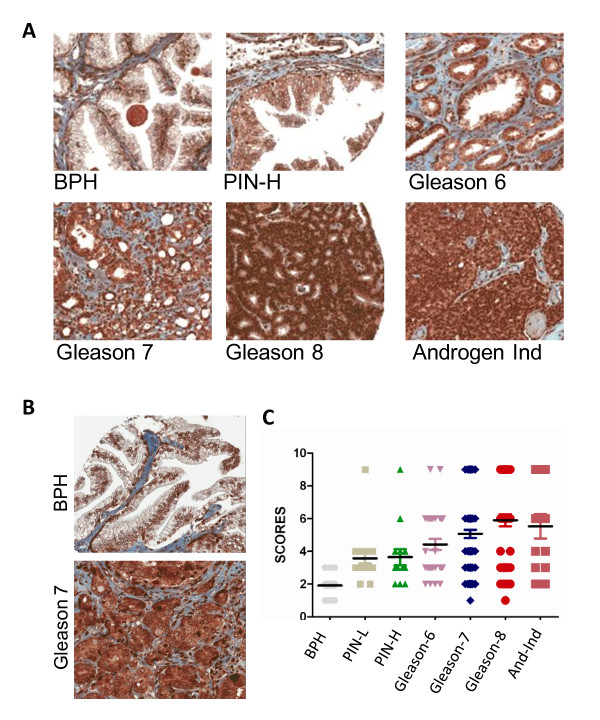
**Strong immunoreactivity for NPRA in human prostate TMA**. (A) A 200× image of a representative sample from each disease stage is shown. (B) Images (400×) of a representative BPH and Gleason 7 are shown. (C) The final NPRA scores for each sample of different stages of PCa are shown. The bar represents the mean sample score for each category of PCa.

The clinical relevance of NPRA expression during human PCa development was examined in BPH, high grade PIN (prostatic intraepithelial neoplasm) and prostatic adenocarcinoma using a human PCa tissue microarray (TMA) containing 240 samples. The TMA samples included BPH (n = 24), low grade prostatic intraepithelial neoplasm (PIN-L) (n = 21), high PIN (PIN-H) (n = 14), prostate carcinoma (PC) with a Gleason score of 6 (n = 33), PC with a Gleason score of 7 (n = 82), PC with a Gleason score of 8 to 10 (n = 51) and androgen-independent (AI) PC (n = 15). The TMA slide was immunostained with a rabbit anti-human NPRA antibody using a Ventana Discovery XT automated system (Ventana Medical Systems, Tucson, AZ) and the data were statistically analyzed. A representative image (200×) of one sample from each disease stage is shown in Figure 2A. The results demonstrate that the majority of epithelial cells in BPH and PIN-L were weakly stained for NPRA, preferentially in the nucleus (Figure 2B) and that the PIN-H samples were weakly to moderately positive for NPRA. Gleason-6 PCa samples exhibited moderate to strong NPRA immunoreactivity. Weak and focal staining of stromal/inflammatory cells was also observed in these samples. In contrast, NPRA staining was uniformly strong and prominent and predominantly localized to the cytoplasm of the tumor cells in Gleason 7-10 and in AI PCa samples (Figure 2B). Stromal/inflammatory cells in these samples also showed moderate NPRA expression.

The TMA slide was scored for intensity and cellularity by an expert pathologist. The final score was classified as: 0, negative; 1-3, weak; 4-6, moderate; and 7-9, strong. Figure 2C shows the distribution of scores in each disease stage. The results show that the mean sample score increased during PCa progression. The Additional file 3, Table S1 displays a median analysis of NPRA expression in the TMA for 240 subjects. Across all 240 subjects, the median score was 4. Additional file 4, Table S2 shows the frequency in each disease group of having a score falling at or below the median and having one above the median. The number of observations in the BPH group with a score >4 was zero, while for Gleason 6, Gleason 7, Gleason 8-10 and AI groups the numbers were respectively 14 (of 33), 43 (of 82), 34 (of 51) and 8 (of 15). A chi-squared (two-way frequency table) value of 50.761 with asymptomatic significance of p <0.0001 was obtained, suggesting that the relationship between NPRA expression and PCa stage is very strong. A Kruskal-Wallis test indicated that the difference in NPRA expression among the seven diagnostic groups was highly significant (p < 0.0001). The pairwise Wilcoxon-Mann-Whitney tests show that NPRA expression is strongly associated with PCa progression. The elevated NPRA expression in high-grade tumors may reflect its role in tumor-stromal interaction. Since the outcomes of the Kruskal-Wallis and Wilcoxon-Mann-Whitney tests are of ordinal value and do not follow the normal distribution that the ANOVA or t-test requires, a nonparametric version of these two methods was used.

### NPRA deficiency impairs engraftment of PCa cells

**Figure 3:**
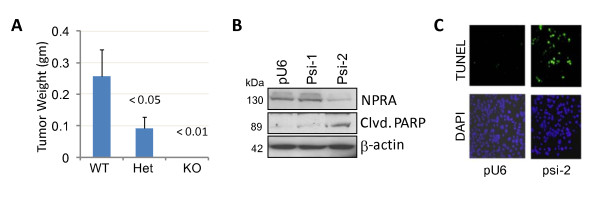
**NPRA-deficiency impairs tumor engraftment and induces apoptosis of PCa cells**. (A) NPRA-deficiency impaired engraftment of TRAMP-C1 cells. Three groups of mice (wild type (WT), heterozygous (Het) and homozygous (NPRA-KO), (n = 5 per group) were injected s. c. in the left and right flanks with 5 × 10^6 ^TRAMP-C1 cells per site. Mice were euthanized ten weeks after injection. Tumors were excised and weighed. Mean tumor weights ± SEM are shown. (B-C) NPRA deficiency induced apoptosis of PCa cells. TRAMP-C1 cells were transiently transfected with psiNPRA (si1 and si2) and control plasmid (pU6). Cells were harvested 72 hrs later and whole cell lysates were analysed for NPRA and PARP by western blotting. (C) TRAMP-C1 cells were transfected with pU6 or psi2 plasmids. Forty-eight hours after transfection, apoptosis was monitored by TUNEL assay.

Since, NPRA signaling is involved in inflammation and the local inflammatory milieu plays a role in PCa development, we reasoned that NPRA might be important for prostate tumor growth. The role of NPRA in modulating PCa progression was tested using TRAMP-C1 cells, which form tumors when grafted subcutaneously into syngeneic C57BL/6 hosts [23]. For *in vivo *assays, C57BL/6 (WT), NPRA-heterozygous (NPRA-het) and NPRA-KO mice were injected subcutaneously with TRAMP-C1 cells. Mice were euthanized seven weeks after injection and tumor sizes and weights were compared (Figure 3A). TRAMP-C1 cells failed to engraft in NPRA-KO mice and no visible tumors were detected in the homozygous group ten weeks after tumor cell injection. Some tumor growth was observed in NPRA-het mice, but at a significantly reduced level compared to that in WT C57BL/6 mice, suggesting that host NPRA gene dosage is a determining factor for the growth of tumor cells in these mice. The role of NPRA deficiency in the survival of TRAMP-C1 cells was tested in vitro by ectopic expression of a plasmid encoding small interfering RNA against NPRA (siNPRA). Expression of siNPRA-2, but not siNPRA-1, significantly reduced expression of NPRA (Figure 3B). Apoptosis was detected by western blotting for PARP cleavage (Figure 3B) and by the terminal transferase dUTP nick end labeling (TUNEL) assay (Figure 3C). Downregulation of NPRA expression by siNPRA-2 induced significant apoptosis in PCa cells.

### NPRA downregulation inhibits MIF expression

**Figure 4:**
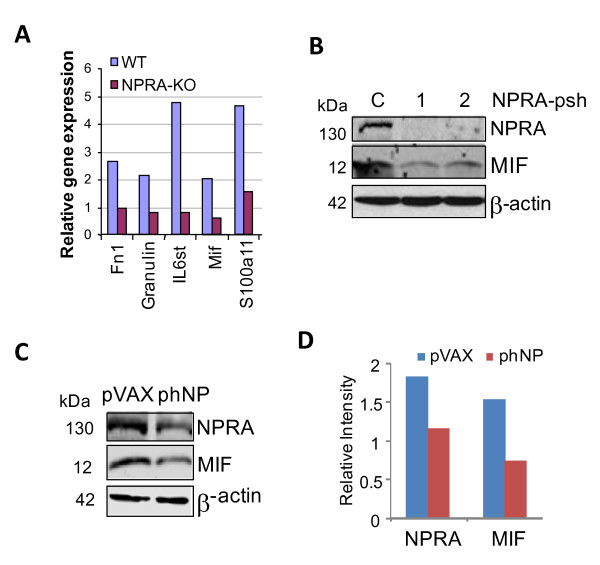
**NPRA depletion inhibits MIF expression**. **(A) **SuperArray analysis of prostate tissues of NPRA-KO and WT C57BL/6 mice. The relative expression level of genes that are altered in the prostate tissues of NPRA-KO vs. WT is shown. (B-C) Cells were transfected with iNPRAs. Whole cell lysates were extracted 72 hrs after transfection and examined for NPRA and MIF by western blotting. TRAMP-C1 cells were transfected with pshNPRAs or empty vector (B); PC3 cells were transfected with phNP_73-102 _plasmid or pVAX plasmid (C). (D) Relative band intensity for NPRA and MIF expression in Fig. 4C is shown.

We reported previously that NPRA-deficient mice fail to mount an inflammatory response, as exemplified by the lack of goblet cell hyperplasia and infiltration of eosinophils in the lungs of NPRA-KO mice compared to those of WT mice, when sensitized and challenged with ovalbumin [19]. The lack of inflammatory response correlated with reduced levels of inflammatory cytokines IL-4, IL-5 and IL-6 in the bronchoalveolar lavage (BAL) fluid of the NPRA-KO mice relative to that of WT mice [19,25]. To examine whether the antitumor effects of iNPRA were due to lack of local inflammation in prostate tissue, we injected mice with lipopolysaccharide (LPS), a potent inducer of local inflammation and compared prostate tissues for alterations in gene expression in WT and NPRA-KO mice. Prostate tissue was collected from LPS-treated and control mice, and total RNA was examined for differential gene expression using a mouse autoimmune and inflammatory response Oligo GEarray (SuperArray, MD). Analysis of genes altered more than two-fold during LPS challenge in WT and NPRA-KO mice identified 24 genes that are either upregulated (15) or downregulated (9) in the prostate tissue of NPRA-KO mice compared to their expression levels in control mice. A few of the genes that are down-regulated during LPS stimulation in NPRA-KO mice is shown in Figure 4A, and include: fibronectin 1 (Fn1), which is involved in the acute phase response [26], granulin [27] and S100 calcium binding protein A 11 (S100a11) [28], which are cytokines, IL6 signal transducer (IL6st; also known as gp130), a cytokine receptor [29,30] and MIF, which is involved in the inflammatory response [31].

Since, MIF has been reported to be involved in PCa progression [31-33], the possibility that NPRA depletion modulates MIF expression was tested using shRNAs for NPRA in TRAMP-C1 cells. As shown in Figure 4B, transfection of TRAMP-C1 cells with shNPRA-1 and shNPRA-2 reduced NPRA expression >80% and also decreased MIF expression >90%. Since overexpression of plasmid-encoded NP_73-102 _downregulates NPRA (Additional file 5, Fig. S3), pNP_73-102 _was also used as an inhibitor of NPRA (iNPRA) in this study. Ectopic expression of the plasmid encoding NP_73-102_, but not the pVAX vector, reduced both NPRA (~40%) and MIF expression (~50%) in PC3 cells (Figure 4C-D) and in TRAMP-C1 cells (data not shown).

### iNPRA reduces tumor burden in part by downregulating MIF

**Figure 5:**
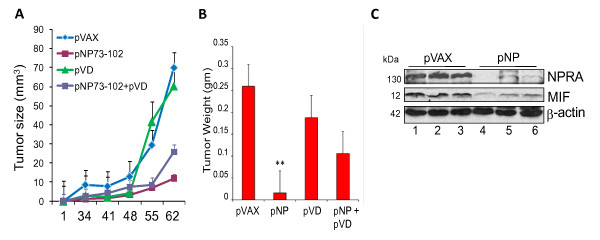
**iNPRA treatment reduces tumor burden by inhibiting MIF expression**. (A-B) Effects of iNPRA in TRAMP-C1 inoculated xenografts in immunocompetent mice. Four groups of C57BL/6 mice (n = 7 per group) were injected s.c. in the right flank with 5 × 10^6 ^TRAMP-C1 cells. Two weeks later, tumor-inoculated mice were treated with CNPs encapsulated with pVAX, pNP_73-102_, pVD or a combination of pNP_73-102 _and pVD i.p. twice a week until euthanized. The tumor size (A) was measured at the indicated days, and the weight was recorded after tumor resection (B). (C) NPRA expression correlates with MIF expression in tumor lysates. Tumor lysates from B were analysed for NPRA and MIF by western blotting. pVAX (lanes 1-3) and pNP_73-102 _(lanes 4-6). β-actin was used as a loading control.

To rule out the possibility that impaired engraftment of TRAMP-C1 cells in NPRA-KO mice is due to immune rejection, we examined the potential of NPRA inhibition to block the growth of TRAMP-C1 cells in immunocompetent C57BL/6 mice. Mice were inoculated with TRAMP-C1 cells and divided into four groups. Two weeks later, mice in each group were injected i.p. twice a week with chitosan nanoparticles (CNPs) encapsulating plasmid DNA (25 μg/mouse) encoding empty vector (pVAX), pNP_73-102_, or a control peptide encoding human vessel dilator (pVD) or a combination of 12.5 μg each of pNP_73-102 _and pVD, using methods as described [19,20]. Mice were monitored for tumor growth and tumor sizes were recorded on the indicated days (Figure 5A). Tumor growth was significantly inhibited in mice treated with pNP7_3-102 _compared to pVAX- or pVD-treated groups. Mice were euthanized on day 65 after treatment, and tumor weights were measured and compared (Figure 5B). As shown in Figure 5A-B, a significant reduction (p < 0.05) in tumor burden was seen in mice treated with 25 μg of pNP_73-102 _but not with the pVAX or pVD plasmids. Mice treated with 12.5 μg pNP_73-102 _showed only moderate inhibition of tumor burden. The plasmid pVD encodes a peptide corresponding to human VD and is not homologous with mouse VD; thus, lack of any antitumor effects in pVD-treated mice suggests the specificity of these peptides in vivo. To understand the antitumor effects of pNP_73-102_, we examined NPRA and MIF expression in TRAMP-C1-engrafted tumor lysates from representative control (pVAX) and pNP_73-102_-treated mice. The results (Figure 5C) show that treatment of mice with pNP_73-102_, but not with pVAX, significantly reduced expression of NPRA and MIF; therefore, expression of these proteins may be linked to growth of primary tumors in TRAMP-C1-inoculated C57BL/6 mice.

**Figure 6:**
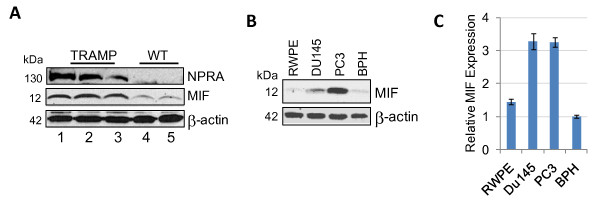
**NPRA and MIF expression in primary prostate tumors and PCa cell lines**. (A) NPRA and MIF expression in primary prostate tumors. Prostate tissues were homogenized using a polytron and cell lysates were analyzed for NPRA and MIF by western blotting. Lanes 1-3: lysates of TRAMP prostates. Lanes 4-5: lysates of C57BL/6 prostates. (B) Whole cell lysates of tumor cell lines and control (normal) cells were analyzed by western blotting. (C) Total RNA of indicated cell lines was analyzed for MIF by real-time PCR.

Lastly, we examined NPRA and MIF expression in primary prostate tumors from TRAMP mice. Western blots showed that NPRA and MIF are detected in the lysates of primary prostate tumors from TRAMP mice of varying ages (18-30 weeks of age) (Figure 6A; lanes 1-4) but not in prostates from age-matched WT C57BL/6 mice (18 and 28 weeks of age) (Figure 6A; lanes 5-6). These results suggest that tumor cell lines, as well as primary prostate tumors of TRAMP mice, show significantly higher levels of NPRA and MIF compared to normal cells or prostate cells from C57BL/6 mice. We also compared NPRA and MIF expression in total cell lysates of human PCa cells by western blotting. Results presented in Figure 6B suggest that increased MIF was seen in the lysates of PC3 and DU145 cells that express NPRA abundantly (Figure 1B) compared to the lysates of BPH and RWPE. MIF protein expression in PC3 and DU145 cells parallelled with mRNA expression, as shown by real-time PCR data (Figure 6C). The results of these studies suggest that NPRA regulates MIF expression in PCa cells.

## Discussion

There remain several overarching challenges in PCa research: the lack of specific clinical markers for early diagnosis and prognosis of PCa and the need to identify drugs that target androgen-independent PCa tumor cells directly without damaging healthy cells. In this study we show that NPRA is a potential biomarker for PCa and candidate for PCa therapy.

One important finding of our study is the demonstration that NPRA is significantly over-expressed in mouse and human PCa cells compared to normal cells. Screening of a human PCa tissue microarray containing 240 tissue samples shows that NPRA is also over-expressed in human tissues including high grade PIN (prostatic intraepithelial neoplasm) and prostatic adenocarcinoma. The benign hyperplastic glands exhibited significantly lower NPRA expression than localized PCas. These data are consistent with our previous report and with the data in this study, showing that NPRA is highly expressed in both human and mouse PCa cell lines and in advanced PCa tissues, but not in a normal prostate epithelial cell line or in a benign prostate hyperplasia epithelial cell line [19,34,35]. It is to be noted that NPRA was expressed in the androgen-dependent cell line LNCaP but not in the stromal cell line, WPMY. However, expression of ANP was detected in culture supernatants of PC3 and DU145 PCa cells and WPMY stromal cells but not in supernatants from normal prostate epithelial cells or LNCaP cells. These results suggest that ANP produced by stromal cells can signal via NPRA on androgen-dependent cells (paracrine), whereas androgen-independent cells produce both ANP and NPRA and can signal in an autocrine manner. Thus, ANP-NPRA signaling may play a key role in engaging PCa cells with stroma during PCa pathogenesis. Hence, PCa may be better managed by inhibiting ANP-NPRA signaling.

Further, we found a significant association between NPRA expression and Gleason score and pathological stage. Results from the tissue array studies show that NPRA is an independent predictor of advanced PCa, and may therefore be useful as a clinical marker. Although, a number of marker antigens have been reported for PCa, none of them is specific enough to pass the clinical test for use in PCa prognosis. Given the strong positive correlation (r = 0.64, p < 0.005) between NPRA expression and the severity of the clinical stage, particularly in androgen-independent PCa, NPRA may prove to be an effective clinical prognostic marker.

Our study also suggests that NPRA may be a drug target for treating PCa. Using the TRAMP-C1 spontaneous PCa model, we demonstrated that NPRA-KO mice, which have normal heart, kidney and vascular function, have no detectable increase in postnatal mortality, do not permit growth of implanted PCa cells and have a normal lifespan of over 24 months. Tumor growth is observed in NPRA-het mice but at a significantly reduced level compared to that in WT C57BL/6 mice, which indicates that host NPRA gene dosage is a determining factor for the growth of tumor cells in mice. This finding is consistent with the reports that atrial natriuretic factor peptides (ANP and VD) inhibit the proliferation of PCa cells *in vitro *and in mice [34]. This is presumably due to the feedback inhibition of NPRA expression caused by high doses of ANP or other natriuretic peptides, such as NP_73-102 _(our data) [18,34,36,37]. Thus, while low doses of these peptides stimulate NPRA signaling, high doses inhibit NPRA signaling and show anticancer effects. In sum, NPRA provides a heretofore undescribed target for PCa. This hypothesis is also supported by the observation that NPRA is an upstream regulator of IL-6, which has been reported as a target for PCa therapy [38,39].

The finding that pNP_73-102 _inhibits NPRA expression prompted us to examine its role in treating PCa. TRAMP-C1 cells injected into C57BL/6 mice induced tumors in the control mice but not in pNP_73-102_-treated mice. These findings demonstrate the potential utility of pNP_73-102 _for the treatment of PCa. Although the mechanism of tumor inhibition by pNP_73-102 _is unknown, the evidence that pNP_73-102 _significantly decreases the expression of NPRA suggests that this may be the explanation for its antitumor effect. A perceived limitation in iNPRA therapy for PCa is the normal physiological role of NPRA in blood pressure regulation. To address this issue we compared blood pressure of NPRA-KO mice with that of TRAMP mice and found no relationship between NPRA expression, blood pressure levels and PCa incidence (Additional file 6, Fig. S4), which is consistent with studies in humans that showed no relationship between blood pressure and PCa [40,41].

Another major finding of our report is that the antitumor effects of limiting NPRA expression may be due to a reduction in inflammation in the tumor environment. Our evidence shows that a number of molecules may be regulated by NPRA signaling including MIF and IL-6, both of which have been implicated in PCa development. Increased MIF mRNA expression and serum MIF levels have been associated with progression of PCa when tumor and benign tissue from matched samples were compared [31,33,42]. Elevated IL-6 levels are found in patients with metastatic PCa and are associated with a poor prognosis [43]. Furthermore, aberrant expression of the IL-6 gene and increased production of IL-6 are associated with advanced bone metastasis and increased morbidity [43-46], as well as resistance to chemotherapy [47]. There are three lines of evidence supporting the idea that NPRA is an upstream regulator of MIF in PCa cells: (i) a 2.5-fold reduction in MIF mRNA was found after LPS treatment of NPRA-KO mice compared to WT mice. (ii) MIF expression was detectable in the prostate tissues of TRAMP mice, but not in WT mice, and (iii) NPRA downregulation reduced MIF expression in cultured TRAMP-C1 cells and xenografts. Consistent with these observations, a PCa tissue array stained for NPRA showed expression of MIF (*data not shown*).

Since intratumoral expression of MIF was correlated with serum IL-6 in patients with non-small cell lung cancer [48] and IL-6 was shown to be one of the potential MIF-regulated genes in DU145 cells [32], we speculate that NPRA signaling may regulate IL-6 in PCa cells via MIF. In support of this hypothesis, we found elevated IL-6 in the serum of TRAMP mice during PCa development (*unpublished observation*). These data support our previously reported studies, where lung tissues of NPRA-KO mice failed to induce IL-6 during OVA-induced inflammatory challenge and showed reduced expression of activated p65- and p50-NF-kB [19,20]. Together, these studies show that NPRA may affect PCa progression by regulating in part MIF and IL-6 expression, both of which have been linked to PCa.

In summary, we demonstrate that increased NPRA expression is strongly associated with progression of human PCa and that NPRA deficiency prevents growth of transplanted PCa cells and inhibits tumor burden in TRAMP mice in part by downregulating MIF in PCa cells.

## Materials and methods

### Materials

Normal prostate epithelial cell line (PrEC) was purchased from Lonza (Allendale). RWPE, WPMY, Tramp-C1 and PC3 cells were purchased from the American Type Culture Collection (Manassas, VA, USA). DU145, PC3, benign prostatic hyperplasia (BPH), LNCaP, C4-2, the rat gastric mucosa cell line (RGM1), P2 and CaP2 were described before [22,24,32,49]. The beta-actin antibody was obtained from Sigma, the PARP antibody from Santa Cruz Biotechnology, and the MIF antibody from Abcam. Lipofectamine 2000 reagent was obtained from Invitrogen.

### NPRA antibody production and purification

Antibody against NPRA was generated by injecting rabbits (New Zealand white) with 400 μg of synthetic NPRA peptide (amino acid 1010-1031 of mouse NPRA protein, which is homologous to rat and human NPRA) conjugated to keyhole limpet hemocyanin (BioSynthesis, Inc., Lewisville TX). Antibody was purified by applying serum to a column of protein A/G agarose (Invitrogen, Carlsbad, CA) equilibrated with 20 mM Tris, pH 7.5, 150 mM NaCl, and eluting with 100 mM citrate, pH 3.0. The eluate was neutralized with 5 M NaOH, glycerol was added to 50% and the purified aliquots were stored at -20°C.

### NPRA antibody competition assay

For determining NPRA antibody titer, a 96 well plate was coated with the non-KLH-conjugated NPRA-specific peptide (amino acids 1010-1031 of mouse NPRA protein) that was used to raise the antibody or an unrelated peptide. Rabbit sera from 6 animals were pooled and purified using a protein A/G sepharose column (Pierce). A serial dilution of the antibody was added to each well of a microtiter plate coated with peptides overnight. For the competition assay, purified antibody was incubated with NPRA-specific peptide on ice for 1 hr and then added to the plate. The plate was washed and developed using HRP-conjugated anti-rabbit IgG (Cell Signaling) and HRP-substrate (R & D Systems). The plate was read at 450 nm using a Synergy H4 plate reader (Biotek). The values presented are means of four wells.

### Cell counting and colony assay

At the indicated times, cells were harvested by trypsinization and viable cell numbers enumerated by trypan blue dye-exclusion. To test colonization potential, TR-C1or TR-C3 cells were plated in 100 mm dishes at 1000 cells/plate. After 3 weeks, the resulting colonies were stained with 0.25% crystal violet, photographed and counted.

### Luciferase reporter assays

PC3 cells were co-transfected with hNP_73-102_, mNP_73-102 _or vector alone (pVAX), reporter plasmid (pNPRA-Luc), and a transfection normalization vector (pRenilla-luc). DNA (0.5-1 μg/10^6 ^cells) was transfected into 60% confluent PC3 cells using lipofectamine (Life Technologies). Forty-eight hours after transfection, the reporter activity was measured with the Dual-Luciferase Reporter assay system (Promega) according to the manufacturer's instructions. Luminescent signals were quantified with the Synergy H4 (Biotek). Reporter assay results were based on data averaged from three replicates.

### Tissue microarray (TMA) staining

A human prostate cancer TMA containing 240 samples, prepared in the histology laboratory of the Moffitt Cancer Center Tissue Core Facility was used to test for expression of NPRA and MIF. The TMA slide was stained using a Ventana Discovery XT automated system (Ventana Medical Systems, Tucson, AZ), according to the manufacturer's protocol. Briefly, slides were deparaffinized on the automated system with EZ Prep solution (Ventana). Following heat-induced antigen retrieval, the slide was incubated with NPRA antibody (1:300) for 32 min and Ventana anti-rabbit or anti-goat secondary antibody for 20 min. The detection system used was the Ventana OmniMap kit, and the slide was then counterstained with hematoxylin and dehydrated.

### TMA data analysis

The TMA slide was scored for intensity and cellularity by an expert pathologist. Positive staining for NPRA was scored into four grades, according to the intensity: 0, 1+, 2+ and 3+. The percentage of NPRA-positive cells was scored into three categories: 1 (0-33%), 2 (34-64%) and 3 (65-100%). The product of the intensity and percentage scores was used as the final score. The final score was classified as: 0, negative; 1-3, weak; 4-6, moderate; and 7-9, strong. A median analysis of NPRA scores and the frequency in each disease group of having a score at or below the median was performed. Also, the chi-squared test, the Kruskal-Wallis test and the Wilcoxon-Mann-Whitney test were used to compare the scores by groups. Comparisons were done for (1) PIN-L vs. BPH; (2) PIN-H vs. BPH; (3) Gleason-6 vs. BPH; (4) Gleason-7 vs. BPH; (5) Gleason-8 to 10 vs. BPH and (6) AI vs. BPH.

### Animals

Male C57BL/6 mice were purchased from the National Cancer Institute. Male C57BL/6 NPRA-KO or NPRA-het were described before [19]. All mice were maintained in a pathogen-free environment and all procedures were reviewed and approved by the University of South Florida Institutional Animal Care and Use Committee.

### Preparation of plasmid nanoparticles and administration to mice

Plasmids encoding NP_73-102_, hNP_73-102 _and VD were constructed as described previously [14,19]. Plasmids encoding siRNAs against NPRA were described previously [19]. Plasmids encoding shNPRAs were purchased from Origene. For transfection, epithelial cells at 60% confluence (log phase) were incubated in complete medium at 37°C with plasmid DNA (1 μg/10^6 ^cells) complexed with lipofectamine (GibcoBRL Life Technologies, Carlsbad, CA). For tumor cell inoculation, TRAMP-C1 cells were trypsinized, washed and resuspended in PBS at 5 × 10^7 ^cells per ml. Mice were injected s.c. in the flank with 100 μL of resuspended cancer cells. For evaluating the effects of iNPRA in modulating tumor progression, plasmids encapsulated in chitosan nanoparticles (25 μg of plasmid plus 125 μg of chitosan) were administered i.p. twice a week until sacrificed. Tumor sizes were measured externally by calipers, and at the end of experiment (day 62), the mice were euthanized and the tumors were removed and weighed. Proteins from tumors were extracted and examined for NPRA and MIF expression by western blotting.

### Blood pressure measurement

Diastolic and systolic pressures of age-matched mice were measured using the CODA noninvasive blood pressure system (Kent Scientific). Briefly, mice were placed in a restrainer on a hot water blanket and the restrainer was covered with a warm water glove. The occlusion cuff was fitted to the base of the tail and the VPR cuff slid down until it reached the occlusion cuff. Maximum occlusion pressure was set to 250 μL with a deflation time of 20 seconds and a minimum volume of blood flow in the tail of 10 μL. The occlusion cuff was inflated to impede the blood flow to the tail. As the occlusion cuff is deflated, a second tail cuff with the VPR sensors records the pressure at the point where blood flow returns. The systolic is measured at the first appearance of tail swelling and the diastolic is calculated when the increasing rate of swelling ceases in the tail.

### Western blot analysis

Western blot assay was performed as previously described [49]. Cells were lysed, total cellular protein (120 μg) was separated by SDS-PAGE, blotted to nitrocellulose, and incubated with antibodies to specific proteins. Bands were visualized by enhanced chemiluminescence (Amersham Life Sciences, Piscataway, NJ) on Kodak X-OMAT-AR film.

### Real-time PCR analysis

Total RNA was isolated using the RNeasy mini kit (Qiagen). One tube cDNA synthesis followed by real-time PCR was performed in a 25 μl reaction mixture using Taqman RNA-to-CT™ *1-Step *Kit (Applied Biosystems). Quantitative real-time PCR was carried out on the CFX96 real-time System (Bio-Rad). Taqman gene expression assays (Applied Biosystems) Hs00418568, Hs00236988 and 4333762, respectively are used for amplification of NPR1, MIF and β-actin. The conditions for the real-time PCR assay were 15 min at 48°C, 10 min at 95°C, 40 cycles of 15 sec at 95°C and 60 sec at 60°C. Expression of each target mRNA relative to β-actin was calculated under experimental and control conditions based on threshold cycle (*C*_t_) as , where Δ*C*_t _= *C*_t target _- *C*_t β-actin _and Δ (Δ*C*_t_) = Δ*C*_t experimental _- *C*_t control_.

### ANP ELISA

Duplicate aliquots of 50 μl of culture supernatants were assayed for ANP concentration using a fluorescent immunoassay kit (Phoenix Pharmaceuticals, Burlingame CA). ANP standards were run to generate a standard curve that was used to calculate the average ANP concentration.

### SuperArray analysis of prostate tissues

NPRA-KO and WT C57BL/6 mice (n = 4) were injected i.p. with LPS (1 mg/kg body weight) for 3 hrs, prior to prostate harvesting. Total RNA was isolated using an RNAeasy kit (QIAGEN, Valencia, CA) and a pool of total RNA by group hybridized to the mouse autoimmune and inflammatory response Oligo GEarray (SuperArray Frederick, MD), according to the manufacturer's instructions. The X-ray films were scanned, and the spots were analyzed using SuperArray Software. The relative expression level was determined by comparing the signal intensity of each gene in the array after normalization to the signal of a set of housekeeping genes.

### Statistics

The number of mice used in each test group was a minimum of four. Experiments were repeated at least once, and measurements were expressed as means ± SD. Pairs of groups were compared through the use of Student's *t *tests. Differences between groups were considered significant at *p *≤ 0.05.

## Abbreviations

ANP: atrial natriuretic peptide; CNP: chitosan NP; het: heterozygous; KO: knockout; MIF: macrophage inhibitory factor; NP: nanoparticles; NPRA: natriuretic peptide receptor A; PCa: prostate cancer; PIN: prostatic intraepithelial neoplasia; siRNA: small interfering RNA; TRAMP: transgenic adenocarcinoma mouse prostate; WT: wild type.

## Competing interests

The authors declare that they have no competing interests.

## Authors' contributions

XW designed the experiments, interpreted the results and prepared the manuscript under the supervision of SM. PR, RP, GH and XK provided technical assistance for experiments. GH also contributed to the manuscript editing. DC provided expertise in scoring TMA slides. KLM and PLD provided expertise in MIF related studies. SSM provided key reagents for the study. SSM, KLM and PLD critically evaluated the manuscript. All authors read and approved the final manuscript.

## Supplementary Material

Additional file 1 **Fig. S1: Characterization of rabbit polyclonal antibody to NPRA**. (A) NPRA competition assay. Reactivity of of anti-NPRA antibody to an unrelated (U) peptide and NPRA-peptide (S) is shown. For the competition assay, NPRA-antibody was adsorbed with NPRA peptide (20 ug/ml) (referred to as S-A) prior to incubation. (B-D) Immunofluorescence (B) and immunohistochemistry (C-D) of anti-NPRA antibody. The indicated cell lines were cultured on chamber slides and immunostained using anti-NPRA Ab. As a negative control, PC3 cells were incubated with secondary Ab alone (Control). (C) Two identical multi-tissue TMA slides containing colon, prostate, breast, and pancreas tumor tissues were used to optimize immunostaining. TMAs slides were incubated with NPRA-Ab (left side) or no antibody (right side). (D) Demonstrate specificity of NPRA antibody. Identical tumor tissues were immunostained with either NPRA antibody (top) or NPRA-antibody adsorbed with NPRA peptide (20 ug/ml). Click here for file

Additional file 2 **Fig. S2: Evaluation of TRAMP tumor cell growth potential and colony-forming ability**. (A) Viability counts of tumor cells after four days. TRAMP-C1, -C2 and -C3 cells were plated at 10^5 ^cells per plate for 4 days and viable cell numbers were enumerated at the indicated days by trypan blue dye-exclusion. (B & C) Tumor cell colony formation after three weeks. TRAMP-C1 or TR-C3 cells were plated in 100 mm dishes at 1000 cells/dish. After 3 weeks, the colonies were stained, photographed (B) or counted (C). Click here for file

Additional file 3 **Table S1: Median analysis of NPRA expression in tissue multi-array from 240 subjects**. Click here for file

Additional file 4 **Table S2: Frequency of Gleason scores above and below the median**. Click here for file

Additional file 5 **Fig. S3: pNP_73-102 _inhibits NPRA expression**. PC3 cells were co-transfected with pVAX, phNP73-102, pVD or pmNP73-102 and pNPRA-luc plasmid and pRenilla-luc plasmids. Forty-eight hrs after transfection, lysates were analyzed for luciferase reporter activity. Relative luciferase activity *± *SD is shown. Click here for file

Additional file 6 **Fig. S4: Blood pressure measurements in NPRA knockout mice compared to wild type and TRAMP mice**. Diastolic and systolic pressure of age-matched wt (n = 3), NPRA-KO (n = 4) and TRAMP (n = 4) male mice were measured using the CODA noninvasive blood pressure system (Kent Scientific). Data is presented as mean pressure ± SD. Click here for file
