# Supplementary figures and images for: Natriuretic Peptide Receptor A as a Novel Target for Prostate Cancer
Source: Mol Cancer. 2011 May 17;10:56. doi: 10.1186/1476-4598-10-56 (PMC3121714; doi:10.1186/1476-4598-10-56)

## Slide 1
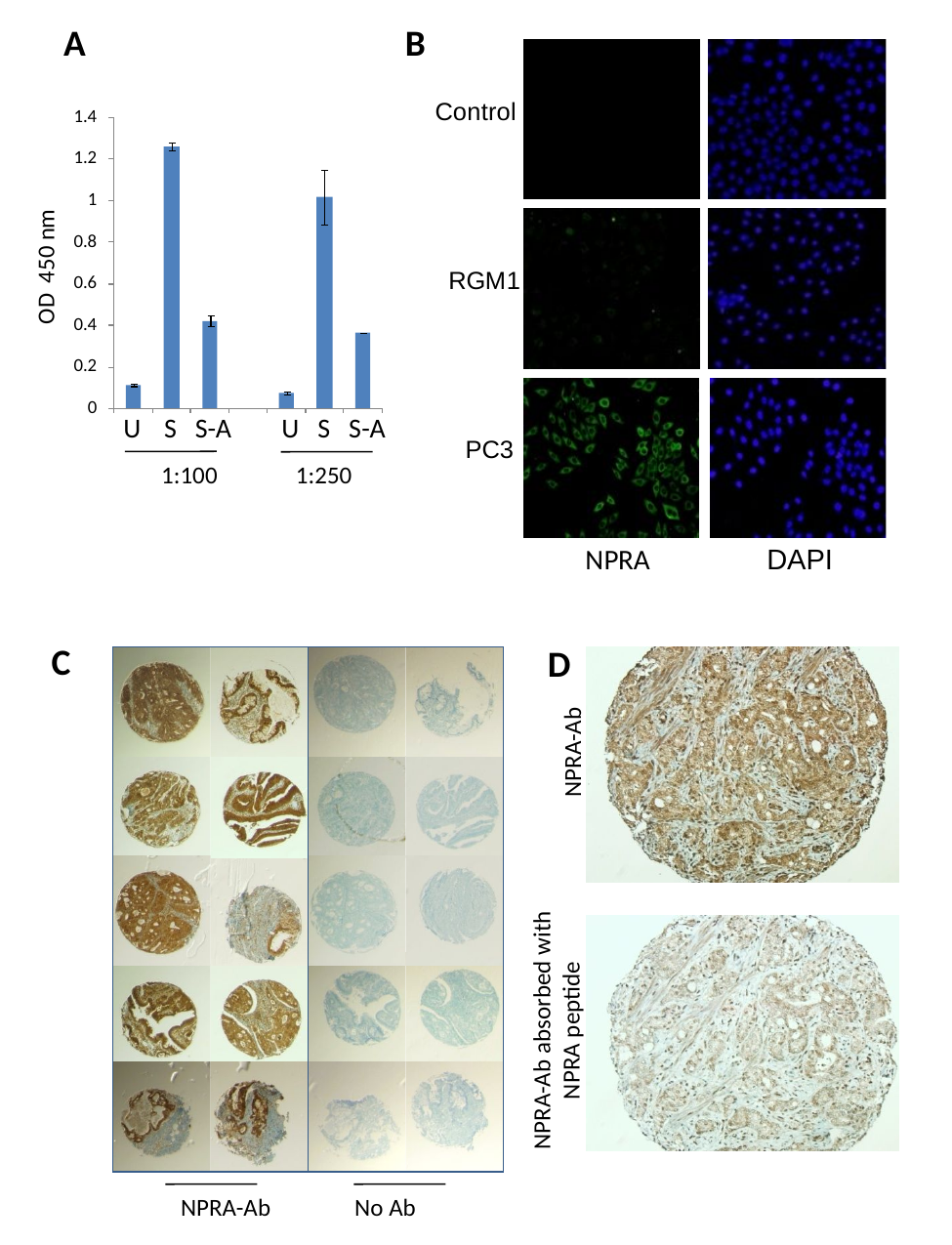

A
B
1:100 1:250
NPRA-Ab
NPRA-Ab absorbed with
NPRA peptide
NPRA-Ab No Ab
C
D

Supplement: Additional file 1 — Fig. S1: Characterization of rabbit polyclonal antibody to NPRA. (A) NPRA competition assay. Reactivity of of anti-NPRA antibody to an unrelated (U) peptide and NPRA-peptide (S) is shown. For the competition assay, NPRA-antibody was adsorbed with NPRA peptide (20 ug/ml) (referred to as S-A) prior to incubation. (B-D) Immunofluorescence (B) and immunohistochemistry (C-D) of anti-NPRA antibody. The indicated cell lines were cultured on chamber slides and immunostained using anti-NPRA Ab. As a negative control, PC3 cells were incubated with secondary Ab alone (Control). (C) Two identical multi-tissue TMA slides containing colon, prostate, breast, and pancreas tumor tissues were used to optimize immunostaining. TMAs slides were incubated with NPRA-Ab (left side) or no antibody (right side). (D) Demonstrate specificity of NPRA antibody. Identical tumor tissues were immunostained with either NPRA antibody (top) or NPRA-antibody adsorbed with NPRA peptide (20 ug/ml). [file 1476-4598-10-56-S1.PPT]

## Slide 1
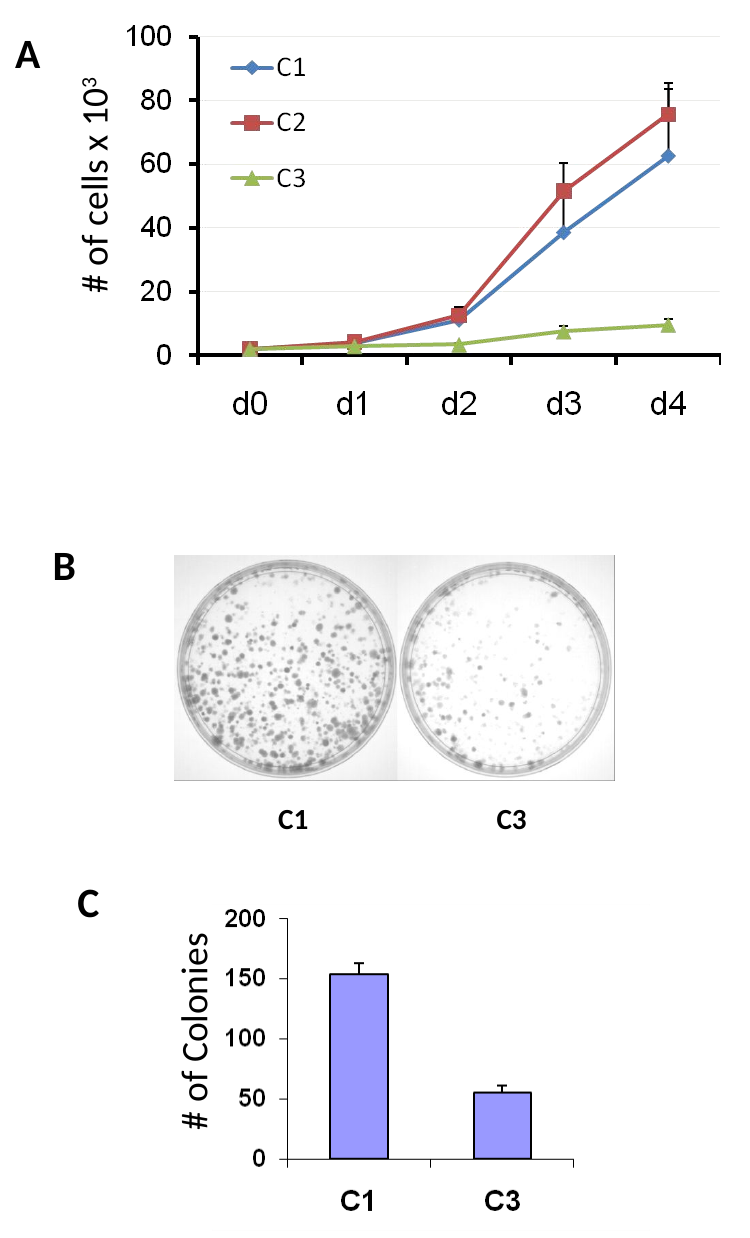

A
# of cells x 103
B
C1 C3
C
# of Colonies

Supplement: Additional file 2 — Fig. S2: Evaluation of TRAMP tumor cell growth potential and colony-forming ability. (A) Viability counts of tumor cells after four days. TRAMP-C1, -C2 and -C3 cells were plated at 105 cells per plate for 4 days and viable cell numbers were enumerated at the indicated days by trypan blue dye-exclusion. (B & C) Tumor cell colony formation after three weeks. TRAMP-C1 or TR-C3 cells were plated in 100 mm dishes at 1000 cells/dish. After 3 weeks, the colonies were stained, photographed (B) or counted (C). [file 1476-4598-10-56-S2.PPT]

## Slide 1
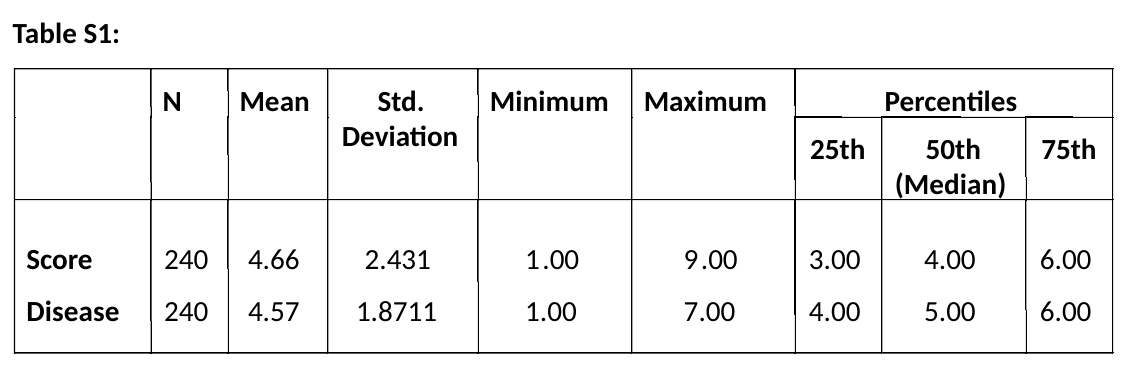

Table S1:
N
Mean
Std.
Minimum
Maximum
Percentiles
Deviation
25th
50th
75th
(Median)
240
4.66
2.431
1
.00
9
.00
3.00
4.00
6.00
Score
240
4.57
1.8711
1.00
7.00
4.00
5.00
6.00
Disease

Supplement: Additional file 3 — Table S1: Median analysis of NPRA expression in tissue multi-array from 240 subjects. [file 1476-4598-10-56-S3.PPT]

## Slide 1
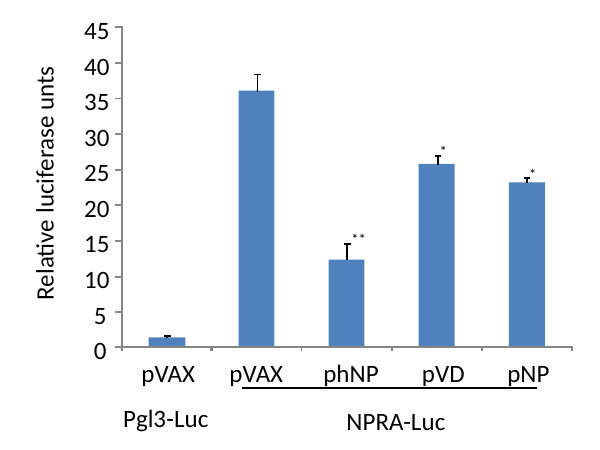

45
40
35
30
*
*
**
25
Relative luciferase unts
20
15
10
5
0
pVAX pVAX phNP
pVD pNP
1
2
3
4
5
Pgl3
-Luc
 NPRA-Luc

Supplement: Additional file 5 — Fig. S3: pNP73-102 inhibits NPRA expression. PC3 cells were co-transfected with pVAX, phNP73-102, pVD or pmNP73-102 and pNPRA-luc plasmid and pRenilla-luc plasmids. Forty-eight hrs after transfection, lysates were analyzed for luciferase reporter activity. Relative luciferase activity ± SD is shown. [file 1476-4598-10-56-S5.PPT]

## Slide 1
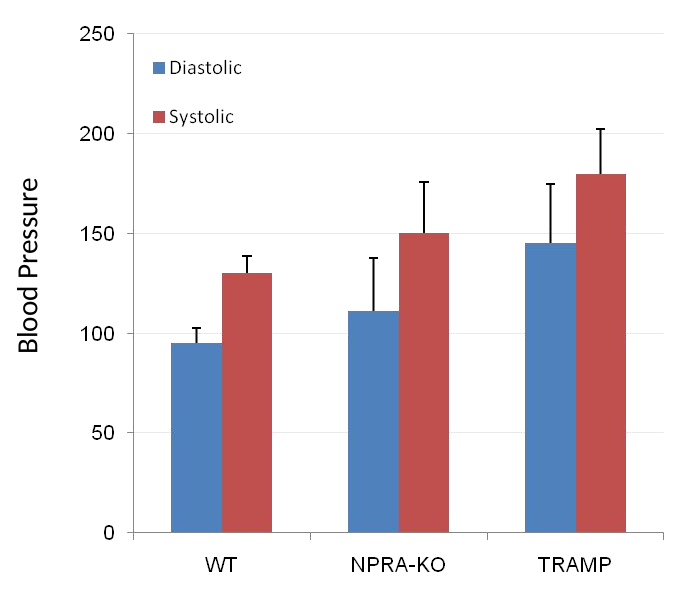

Blood Pressure

Supplement: Additional file 6 — Fig. S4: Blood pressure measurements in NPRA knockout mice compared to wild type and TRAMP mice. Diastolic and systolic pressure of age-matched wt (n = 3), NPRA-KO (n = 4) and TRAMP (n = 4) male mice were measured using the CODA noninvasive blood pressure system (Kent Scientific). Data is presented as mean pressure ± SD. [file 1476-4598-10-56-S6.PPT]
