# Supplementary material for: Natriuretic Peptide Receptor A as a Novel Target for Prostate Cancer
Source: Mol Cancer. 2011 May 17;10:56. doi: 10.1186/1476-4598-10-56 (PMC3121714; doi:10.1186/1476-4598-10-56)
Supplement: Additional file 4 — Table S2: Frequency of Gleason scores above and below the median. [file 1476-4598-10-56-S4.PPT]

## Slide 1
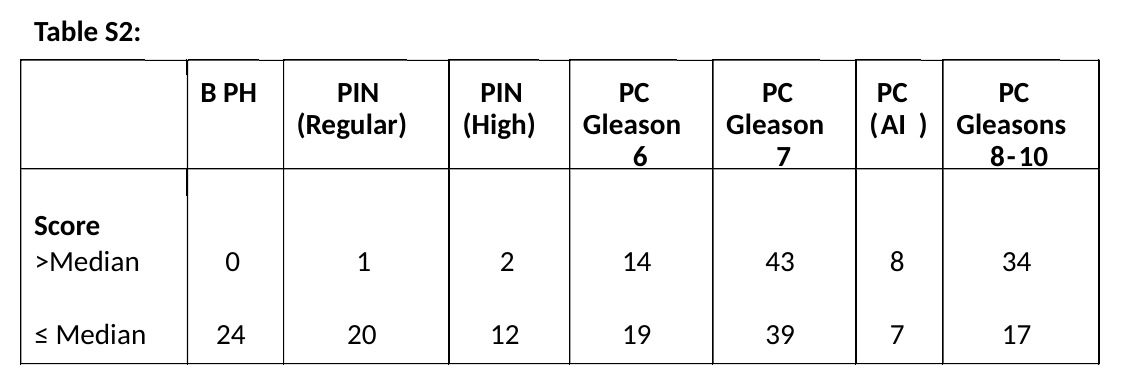

Table S2:
B
PH
PIN
PIN
PC
PC
PC
PC
(Regular)
(High)
Gleason
Gleason
(
AI
)
Gleasons
6
7
8
-
10
Score
>Median
0
1
2
14
43
8
34
≤ Median
24
20
12
19
39
7
17
